# Distressed Communities Index in Patients Undergoing Transcatheter Aortic Valve Implantation in an Affluent County in New York

**DOI:** 10.1155/2021/8837644

**Published:** 2021-08-24

**Authors:** Thomas Bilfinger, Allison Nemesure, Robert Pyo, Jonathan Weinstein, Giridhar Korlipara, Daniel Montellese, Shamim Khan, Neal Patel, Henry Tannous, Ting-Yu Wang, Ely Gracia, Susan Callahan, Puja B. Parikh

**Affiliations:** ^1^Division of Cardiothoracic Surgery, Department of Medicine, State University of New York at Stony Brook, Stony Brook, NY, USA; ^2^Division of Cardiovascular Medicine, Department of Medicine, State University of New York at Stony Brook, Stony Brook, NY, USA

## Abstract

**Background:**

The clinical impact of the distressed communities index (DCI), a composite measure of economic well-being based on the U.S. zip code, is becoming increasingly recognized. Ranging from 0 (prosperous) to 100 (distressed), DCI's association with cardiovascular outcomes remains unknown. We aimed to study the association of the DCI with presentation and outcomes in adults with severe symptomatic aortic stenosis (AS) undergoing transcatheter aortic valve intervention (TAVR) in an affluent county in New York.

**Methods:**

The study population included 286 patients with severe symptomatic AS or degeneration of a bioprosthetic valve who underwent TAVR with a newer generation transcatheter heart valve (THV) from December 2015 to June 2018 at an academic tertiary medical center. DCI for each patient was derived from their primary residence zip code. Patients were classified into DCI deciles and then categorized into 4 groups. The primary and secondary outcomes of interest were 30-day, 1-year, and 3-year mortality, respectively.

**Results:**

Among 286 patients studied, 26%, 28%, 28%, and 18% were categorized into DCI groups 1–4, respectively (DCI <10: *n* = 73; DCI 10–20: *n* = 81; DCI 20–30: *n* = 80; DCI >30: *n* = 52). Patients in group 4 were younger with worse kidney function compared to patients in groups 1 and 2. They also had smaller aortic annuli and were more likely to receive a smaller THV. No significant difference in hospital length of stay or distribution of in-hospital, 30-day, 1-year, and 3-year mortality was demonstrated.

**Conclusions:**

While the DCI was associated with differences in the clinical and anatomic profile, it was not associated with differences in clinical outcomes in this prospective observational study of adults undergoing TAVR suggesting that access to care is the likely discriminator.

## 1. Introduction

The success of new technology can be measured by the time it takes to penetrate a given market. With high research and development costs, adaptation of new technology is typically occurring along socioeconomic gradients [1]. The clinical impact of the distressed communities index (DCI), a composite measure of economic well-being based on the U.S. zip code, is becoming increasingly recognized. Ranging from 0 (prosperous) to 100 (distressed), DCI's association with cardiovascular outcomes remains unknown [2]. Transcatheter aortic valve intervention (TAVR), which was introduced over a decade ago, has radically changed how we treat severe symptomatic aortic stenosis (AS) and bioprosthetic aortic valve degeneration [3]. While data are emerging that TAVR programs are concentrated in wealthier areas, raising questions about access, no data exist on the association between the DCI and outcomes following TAVR. Accordingly, we aimed to study the association of the DCI with presentation, management, and outcomes in adults with severe symptomatic AS or bioprosthetic valve degeneration undergoing TAVR at an academic tertiary care center in an affluent county in New York.

## 2. Methods

We prospectively included all patients undergoing TAVR at Stony Brook University Hospital, an academic tertiary medical center, in our institutional registry. Adults (age >18 years) with severe symptomatic AS and/or failure of a bioprosthetic valve and undergoing TAVR from December 2015 to June 2018 were included in this study.

Suffolk County is the easternmost county on Long Island, New York. It comprises 107 zip codes, each of which comprises at least 500 inhabitants [4]. The DCI is available for all zip codes with more than 500 residents, which captures 99% of the U.S. population. It is a composite score based on 7 metrics: no high-school degree, housing vacancy rate, adults not working, poverty rate, median income ratio, change in employment, and change in business establishments [2]. The 7 evenly weighted variables are used to calculate a zip code rank compared with its geographic peers and then normalized to obtain a raw distress score that ranges from 0 (no distress) to 100 (severe distress). The 7 socioeconomic status (SES) indicators were obtained from 5-year estimates from the 2014 American Community Survey and the Census Bureau County and ZIP Code Business Pattern. The Economic Innovation Group provides a heat map of DCI scores across the U.S. The number of valves estimated in patients with home zip codes in Suffolk was obtained from Medicare claims data for all 107 Suffolk County zip codes.

Patients were classified into deciles of the DCI based on the zip code of their primary address. For analysis, we grouped DCI values > 30 together as there were few patients in each decile above 30. Demographic and medical history extracted included age, sex, body mass index (BMI), prior coronary artery bypass graft surgery (CABG), prior aortic valve replacement (AVR), prior balloon aortic valvuloplasty (BAV), prior mitral valve surgery, prior myocardial infarction (MI), atrial fibrillation (AF), prior pacemaker/defibrillator, prior stroke/transient ischemic attack, chronic obstructive pulmonary disease (COPD), obstructive sleep apnea, diabetes mellitus, carotid disease, peripheral arterial disease, and serum creatinine (mg/dl). The Society of Thoracic Surgeons (STS) predicted the risk of mortality was obtained for each patient. Echocardiographic data extracted included aortic valve area (AVA) and index (AVAI) and left ventricular ejection fraction (LVEF). Aortic annular area and perimeter were also obtained from gated computed tomography angiography (CTA). Procedural data (e.g., access, anesthesia, transcatheter valve size and type, predilatation, and postdilatation) and discharge data (e.g., discharge antiplatelets, discharge anticoagulants, discharge location, length of stay (LOS), and in-hospital and 30-day outcomes (e.g., all-cause mortality, disabling stroke, new pacemaker, and hospital readmission), as well as 1- and 3-year all-cause mortality, were also collected. This study was approved by our Institutional Review Board. A waiver of consent to use data prospectively was obtained for all patients.

Continuous variables were presented as means ± standard deviation (SD) and compared using one-way ANOVA. Categorical variables were presented as percentages and compared with the chi-squared test. Histogram of the DCI was performed for the Suffolk County population, our overall study population, and for patients requiring pacemaker, hospital readmission, and/or discharge to a skilled nursing facility. SPSS version 23.0 (SPSS Inc., Chicago, IL) was used for data analysis, and a two-tailed *P* value of 0.05 was regarded as statistically significant.

## 3. Results

**Figure 1:**
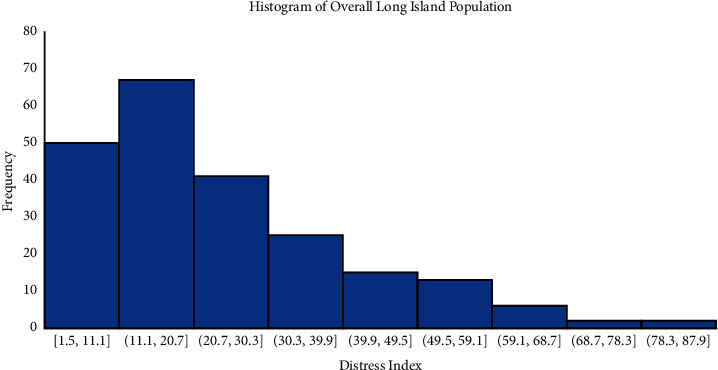
Histogram of the distressed communities index across Long Island, New York.

**Figure 2:**
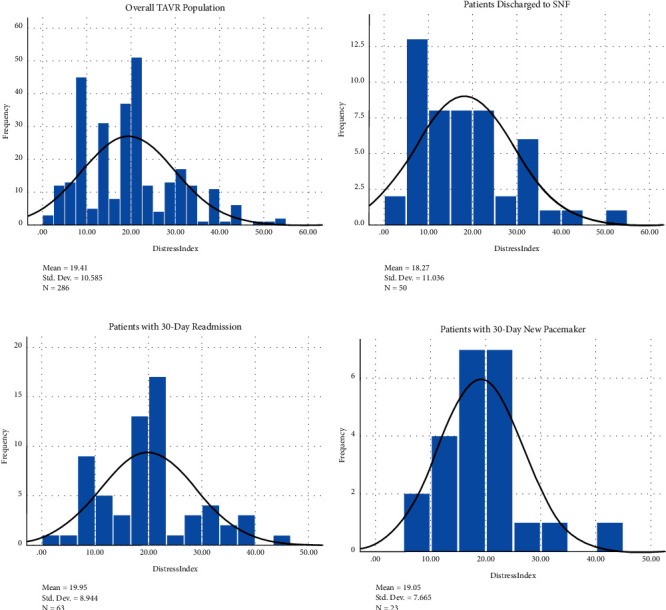
Histogram of the distressed communities index of our study population for TAVR patients (a) overall, (b) requiring a skilled nursing facility, (c) requiring a new pacemaker, and (d) presenting with 30-day readmission.

**Figure 3:**
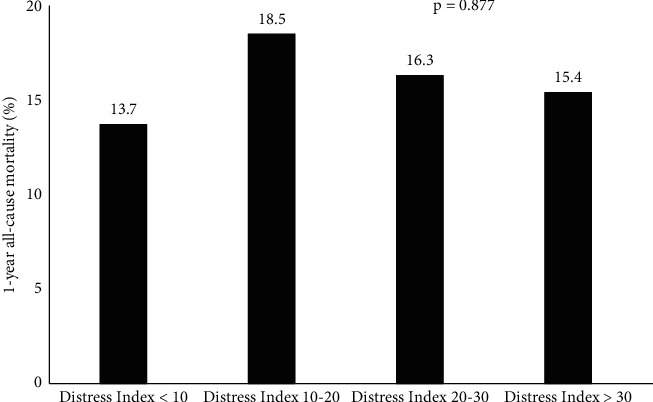
Rates of 1-year all-cause mortality categorized by the distressed communities index group.

The distribution of DCI scores on Long Island, New York, is skewed towards a low DCI score (Figure 1). Our study population included 286 consecutive patients who underwent TAVR from December 2015 to June 2018 at a single academic tertiary care institution. Among the 286 patients studied, 26%, 28%, 28%, and 18% were categorized into DCI groups 1–4, respectively (DCI <10: *n* = 73; DCI 10–20: *n* = 81; DCI 20–30: *n* = 80; DCI >30: *n* = 52). Patients in group 4 had increased serum creatinine compared to patients in groups 1 and 2 (Table 1). They also had smaller aortic annuli and were more likely to receive a smaller THV (Tables 2 and 3). No difference in age and STS predicted risk of mortality was noted across the 4 groups. With respect to outcomes, no significant difference in hospital length of stay or rate of in-hospital and 30-day mortality, stroke, new pacemaker, and readmission was detected (Table 4). The distribution of patients requiring a skilled nursing facility, new pacemaker, or 30-day readmission followed the DCI histogram of that of the overall TAVR population implanted (Figures 2(a)–2(d)). One-year all-cause mortality rates were also similar among the groups (Figure 3), and at 3 years, 76/286 (26.6%) had died.

## 4. Discussion

In this contemporary prospective study of adults undergoing TAVR at an academic medical center in an affluent county in New York, several findings are noteworthy. First, patients with worse DCI were younger, had worse kidney function, and were more likely to receive a smaller THV. Second, DCI was not associated with early in-hospital or 30-day outcomes. Finally, 1- and 3-year mortality was similar across the DCI groups. To our knowledge, this is the first study to assess the association between the DCI and outcomes in patients undergoing TAVR, during a time period where TAVR surpassed SAVR in New York state (Table 5). A likely explanation for the absence of postprocedural differences is the intense competition of regional TAVR programs which all perform in or near the top on the composite metric for benchmarking recently published [5].

The association between socioeconomic status and health outcomes, particularly with cardiovascular disease, has been well documented [6]. Low SES has been linked with a higher prevalence of multiple cardiovascular conditions, including AS [7], rheumatic heart disease [8], and abdominal aortic aneurysm (AAA) [9], as well as worse morbidity and/or mortality in the setting of aortic aneurysm [10], aortic dissection [11], stable coronary artery disease [12], and acute coronary syndromes [13, 14]. Low SES has also been associated with delayed and/or absent referral for cardiac procedures [15, 16] as well as poor outcomes following multiple cardiovascular interventions including PCI [17–19], CABG [20–22], aortic and/or mitral valve surgery [21, 23–25], infrainguinal bypass [26, 27], and aortic aneurysm repair [28–30].

Few studies have examined the association of SES with referral for TAVR referral and/or outcomes [31, 32]. One study examining data from the New York State Department Statewide Planning and Research Cooperative System demonstrated that the proportion of TAVR procedures performed in patients from low-income areas increased over time while that in high-income areas decreased over time, suggesting a resolution of health disparities over time due to penetration of the new technology [31]. Nathan A. et al. recently presented data, however, which suggest that TAVR programs may be concentrated in wealthier areas raising questions about access (Nathan A. et al. Stable Ischemic Heart Disease and TAVR. Presented at the Society for Cardiovascular Angiography and Interventions Scientific Session; April 28–May 1, 2021 (virtual meeting)).

DCI has been associated with higher rates of postoperative morbidity and mortality following CABG [33, 34]. One study demonstrated that, for every 25-point increase in the DCI, the risk-adjusted mortality following CABG increased 14% [34]. While our study did not demonstrate any association between the DCI and outcomes in patients undergoing TAVR, there was a higher rate of high-risk features in the highest DCI group, including worse renal function and smaller aortic annuli.

Our study had a number of limitations. First, observational data in this study were not centrally adjudicated but rather internally validated. Second, our study comprised patients undergoing TAVR, and so, patients who were not referred for TAVR were not captured. Third, the distribution of the DCI in Suffolk County is not generalizable to other regions as Suffolk County, along with its neighboring county of Nassau, is among the most affluent areas in the state of New York. As a result, we are unable to compare differences in truly distressed (i.e., DCI >80) versus prosperous (i.e., DCI <20) areas, and so, we were only able to compare the most affluent versus the least affluent areas of the region. Fourth, ethnic and racial data were not captured in this study [35–38]. The uneven distribution with which TAVR reaches certain racial and ethnic groups has been widely acknowledged [38, 39]. Finally, there may be unknown confounding variables contributing to the associations reported in this study, and the numbers are small so that a type II error cannot be completely excluded.

When innovative technologies (i.e., TAVR) penetrate a specific population, the relationship between socioeconomic disparities and health outcomes can be variable. This prospective observational study of adults with severe symptomatic AS or bioprosthetic aortic valve degeneration suggests that once detected and referred for TAVR, differences in outcomes across the DCI remain similar rendering access and case selection, the likely discriminators.

## Figures and Tables

**Table 1:** Baseline medical history.

	Distress index <10 (*n* = 73)	Distress index 10–20 (*n* = 81)	Distress index 20–30 (*n* = 80)	Distress index >30 (*n* = 52)
Age (years)	80 ± 9	81 ± 8	79 ± 8	77 ± 10
Female gender	36 (49.3%)	35 (43.2%)	35 (43.8%)	32 (61.5%)
Weight (kg)	83 ± 19	80 ± 19	82 ± 21	77 ± 18
Height (meters)	1.67 ± 0.11	1.68 ± 0.10	1.66 ± 0.11	1.63 ± 0.11
Body mass index (kg/m^2^)	29.5 ± 5.8	28.0 ± 6.6	29.3 ± 6.4	27.6 ± 8.3
STS predicted risk of mortality (PROM)	6.4 ± 5.7	6.4 ± 4.5	6.7 ± 4.2	6.1 ± 5.4
Serum creatinine (mg/dl)	1.1 ± 0.5	1.3 ± 1.0	1.2 ± 0.6	1.6 ± 1.6
Prior coronary artery bypass grafting	18 (24.7%)	18 (22.2%)	16 (20.0%)	4 (7.7%)
Prior myocardial infarction	8 (11.0%)	23 (28.4%)	23 (28.7%)	12 (23.1%)
Prior aortic value replacement	1 (1.4%)	2 (2.5%)	7 (8.8%)	3 (5.8%)
Prior balloon aortic valvuloplasty	1 (1.4%)	4 (5.0%)	1 (1.3%)	3 (5.8%)
Prior mitral valve surgery	3 (4.1%)	0 (0%)	7 (8.8%)	0 (0%)
Prior pacemaker/defibrillator	12 (16.4%)	7 (8.6%)	13 (16.3%)	4 (7.7%)
Atrial fibrillator	33 (45.2%)	33 (40.7%)	28 (35.0%)	12 (23.1%)
Chronic obstructive pulmonary disease	12 (16.4%)	20 (24.7%)	19 (23.8%)	10 (19.2%)
Obstructive sleep apnea	6 (8.2%)	9 (11.1%)	8 (10.0%)	2 (3.8%)
Prior stroke/transient ischemic attack	11 (15.1%)	17 (21.0%)	12 (15.0%)	5 (9.6%)
Carotid disease	16 (21.9%)	20 (24.7%)	21 (26.3%)	11 (21.2%)
Peripheral arterial disease	4 (5.5%)	7 (8.6%)	6 (7.5%)	7 (13.5%)
Diabetes mellitus	25 (34.2%)	34 (42.0%)	33 (41.8%)	20 (38.5%)

**Table 2:** Clinical testing.

	Distress index <10 (*n* = 73)	Distress index 10–20 (*n* = 81)	Distress index 20–30 (*n* = 80)	Distress index >30 (*n* = 52)	*P* value
Echocardiogram					
Aortic valve area (cm^2^)	0.71 ± 0.17	0.71 ± 0.20	0.77 ± 0.21	0.76 ± 0.19	0.146
Aortic valve area index (cm^2^/m^2^)	0.38 ± 0.10	0.38 ± 0.11	0.45 ± 0.39	0.43 ± 0.12	0.134
Left ventricular ejection fraction (%)	56 ± 17	54 ± 14	54 ± 15	58 ± 15	0.623

Gated computed tomography					
Aortic annulus area (mm^2^)	447 ± 75	468 ± 95	440 ± 101	408 ± 109	0.010
Aortic annulus perimeter (mm)	77 ± 10	79 ± 9	77 ± 9	73 ± 11	0.018

**Table 3:** Procedural information.

	Distress index <10 (*n* = 73)	Distress index 10–20 (*n* = 81)	Distress index 20–30 (*n* = 80)	Distress index >30 (*n* = 52)
Conscious sedation	64 (87.7%)	61 (75.3%)	61 (77.2%)	37 (71.2%)
Transfemoral access	73 (100.0%)	81 (100.0%)	79 (98.8%)	52 (100.0%)
Predilatation	61 (83.6%)	65 (80.2%)	52 (65.0%)	35 (67.3%)
Transcatheter value type				
Edwards SAPIEN 3	58 (79.5%)	65 (80.2%)	59 (73.8%)	42 (80.8%)
Medtronic Evolut R/PRO	15 (20.5%)	16 (19.8%)	21 (26.3%)	10 (19.2%)
Transcatheter valve size				
20 mm	5 (6.8%)	2 (2.5%)	2 (2.5%)	6 (11.5%)
23 mm	20 (27.4%)	23 (28.4%)	25 (31.3%)	21 (4.4%)
26 mm	33 (45.2%)	30 (37.0%)	31 (38.8%)	20 (38.5%)
29 mm	14 (19.2%)	25 (30.9%)	19 (23.8%)	2 (3.8%)
34 mm	1 (1.4%)	1 (1.2%)	3 (3.8%)	3 (5.8%)
Postdilatation	3 (4.1%)	4 (4.9%)	5 (6.3%)	1 (1.9%)

**Table 4:** Discharge information.

	Distress index <10 (*n* = 73)	Distress index 10–20 (*n* = 81)	Distress index 20–30 (*n* = 80)	Distress index >30 (*n* = 52)	*P* value
Discharge prescriptions					
Aspirin	70 (95.9%)	71 (89.9%)	76 (96.2%)	49 (96.1%)	0.254
Clopidogrel	44 (60.3%)	49 (62.0%)	49 (62.0%)	35 (68.6%)	0.806
Ticagrelor	0 (0%)	2 (2.5%)	1 (1.3%)	4 (7.8%)	0.038
Warfarin	16 (21.9%)	21 (26.6%)	14 (17.7%)	10 (19.6%)	0.576
Apixaban	6 (8.2%)	5 (6.3%)	9 (11.4%)	1 (2.0%)	0.239
Rivaroxaban	2 (2.7%)	1 (1.3%)	2 (2.5%)	1 (2.0%)	0.923
Dabigatran	3 (4.1%)	1 (1.3%)	3 (3.8%)	0 (0%)	0.370
Discharge location					0.545
Home	58 (79.5%)	63 (79.7%)	69 (87.3%)	42 (82.4%)	
Skilled nursing facility	15 (20.5%)	16 (20.3%)	10 (12.7%)	9 (17.6%)	
Length of stay (days)					
Admission to discharge	5.1 ± 5.3	5.7 ± 6.6	5.7 ± 5.1	6.2 ± 6.7	0.776
TAVR procedure to discharge	3.1 ± 2.1	8.0 ± 41.1	3.2 ± 2.6	3.3 ± 4.0	0.424
In-hospital					
Major adverse cardiac events	4 (5.5%)	10 (12.3%)	7 (8.8%)	3 (5.8%)	0.402
All-cause mortality	0 (0%)	2 (2.5%)	1 (1.3%)	1 (1.9%)	0.609
Disabling stroke	2 (2.7%)	1 (1.2%)	0 (0%)	0 (0%)	0.329
New pacemaker	2 (3.3%)	8 (10.8%)	6 (9.0%)	2 (4.2%)	0.282
30-day (includes in-hospital outcomes)					
Major adverse cardiac events	4 (5.5%)	14 (17.3%)	10 (12.5%)	4 (7.7%)	0.102
All-cause mortality	0 (0%)	2 (2.5%)	1 (1.3%)	2 (3.8%)	0.397
Disabling stroke	2 (2.8%)	2 (2.5%)	1 (1.3%)	0 (0%)	0.643
New pacemaker	2 (3.3%)	11 (15.5%)	8 (12.1%)	2 (4.3%)	0.055
All-cause readmission	11 (15.3%)	21 (26.9%)	21 (26.6%)	10 (20.0%)	0.267

**Table 5:** TAVR/SAVR in New York state.

		SAVR			TAVR		
Year	Total	SAVR total	Elective	Urgent/emergent	TAVR total	Elective	Urgent/emergent
2015	5990	3856 (64.4%)	2553 (66.2%)	1303 (33.8%)	2134 (35.6%)	1455 (68.2%)	679 (31.8%)
2016	7094	3746 (67.6%)	2531 (67.6%)	1215 (32.4%)	3348 (47.2%)	2451 (73.2%)	897 (26.8%)
2017	7074	3101 (43.8%)	2156 (69.5%)	945 (30.6%)	3973 (56.2%)	2988 (75.2%)	985 (24.8%)
2018	6911	2841 (41.1%)	1912 (67.3%)	929 (32.7%)	4070 (58.9%)	3166 (77.8%)	904 (22.2%)

## Data Availability

The distressed communities index can be found at eig.org, the Suffolk County demographic data can be found at newyork-demographics.com, and state wide TAVI data can be obtained from the SPARCS database.
